# Fenofibrate therapy and risk of heart failure outcomes in patients with Type 2 diabetes: a propensity-matched cohort study

**DOI:** 10.1093/ehjcvp/pvaf053

**Published:** 2025-07-21

**Authors:** Ji Yoon Kim, Nam Hoon Kim, Jiyoon Lee, Dong-Hoon Kim, Sin Gon Kim

**Affiliations:** Division of Endocrinology and Metabolism, Department of Medicine, Samsung Medical Center, Sungkyunkwan University School of Medicine, Seoul 06351, Republic of Korea; Department of Internal Medicine, Korea University College of Medicine, Seoul 02841, Republic of Korea; Department of Internal Medicine, Korea University College of Medicine, Seoul 02841, Republic of Korea; Department of Biostatistics, Korea University College of Medicine, Seoul 02841, Republic of Korea; Department of Pharmacology, Korea University College of Medicine, Seoul 02841, Republic of Korea; Department of Internal Medicine, Korea University College of Medicine, Seoul 02841, Republic of Korea

**Keywords:** Fenofibrate, Statin, Heart failure, Cardiovascular outcome, Type 2 diabetes

## Abstract

**Aims:**

This study investigated the association between fenofibrate use and outcomes of heart failure (HF) in patients with Type 2 diabetes (T2D).

**Methods and results:**

In a nationwide cohort database (2008–22) in South Korea, patients with T2D (≥30 years) receiving statin therapy were 1:1 matched by propensity score into a statin plus fenofibrate group (*n* = 11 722) and statin only group (*n* = 11 722). The primary outcomes were hospitalization for HF (HHF) and a composite of HHF or cardiovascular death. A Cox proportional hazards model was used to assess the association between treatments and outcomes. During a median of 50.4 months, the incidence rate per 1000 person-years of HHF was 3.44 and 4.13 in the statin plus fenofibrate and statin only groups, respectively (adjusted hazard ratio [HR], 0.80; 95% confidence interval [CI], 0.65–0.98). The adjusted HR for the composite outcome of HHF or cardiovascular death was 0.79 (95% CI, 0.65–0.96). Sensitivity analyses limited to individuals with ≥80% adherence showed consistent results (HHF: adjusted HR, 0.63; 95% CI, 0.43–0.92; composite outcome: adjusted HR, 0.68; 95% CI, 0.48–0.97).

**Conclusion:**

In this propensity-matched cohort study, the addition of fenofibrate to statins was associated with significantly lower risks of HHF and the composite outcome of HHF or cardiovascular death in patients with T2D, suggesting a novel cardiovascular benefit of fenofibrate.

## Graphical Abstract

**Graphical Abstract:**
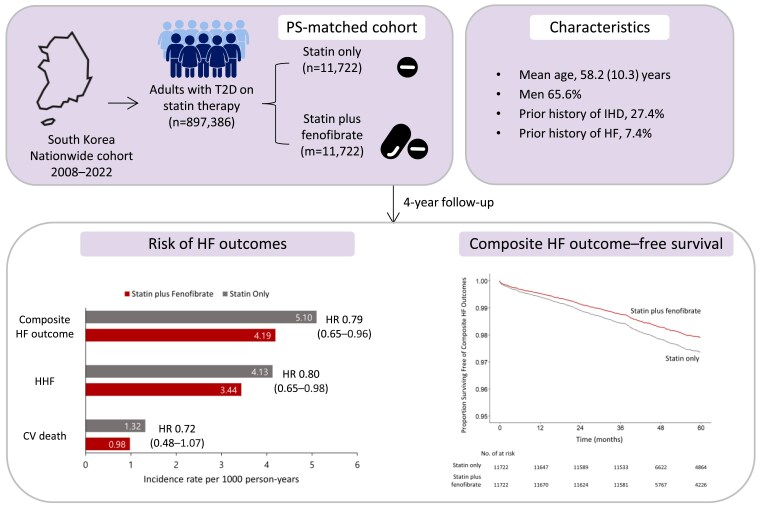

## Introduction

Type 2 diabetes (T2D) is a significant risk factor for the development of heart failure (HF), increasing the risk by two to five times compared with individuals without diabetes.^1-4^ Approximately 44% of patients hospitalized for HF have diabetes.^5^ Heart failure and T2D share several underlying pathophysiological mechanisms, including insulin resistance and chronic inflammation, which create a detrimental cycle.^6^ Diabetes can directly impair cardiac function through oxidative stress, inflammation, lipotoxicity, and mitochondrial dysfunction, leading to a distinct entity known as diabetic cardiomyopathy.^7,8^

Altered lipid metabolism plays a significant role in the development of cardiovascular diseases, including HF, in patients with T2D. Statin therapy has been reported to potentially reduce the risk of hospitalization for HF (HHF) among individuals with HF in a randomized controlled trial^9^ and a meta-analysis.^10^ Diabetic dyslipidaemia, characterized by high triglycerides, low HDL cholesterol (HDL-C), and increased small dense LDL particles, is commonly observed in T2D and is an early event in the development of cardiovascular complications.^11-15^ Elevated triglyceride levels have been independently associated with an increased risk of developing HF.^16^ A prospective study demonstrated that higher triglyceride levels correlated with a greater risk of incident HF, even after adjusting for other cardiovascular risk factors.^17^ Triglyceride-rich lipoproteins, such as VLDL, can directly impair cardiac function and promote adverse myocardial remodelling.^18^

Fenofibrate, a peroxisome proliferator-activated receptor alpha (PPAR-α) agonist, exhibits potent lipid-modifying effects, including reduction of triglycerides and elevation of HDL-C levels.^19^ Fenofibrate has demonstrated possible beneficial effects on HF-related outcomes. In the ACCORD trial, fenofibrate, compared with placebo, reduced the composite outcome of HHF or cardiovascular death by 18% in people with T2D receiving simvastatin.^20^ However, the reduction in HHF alone was not statistically significant, and the beneficial effects of fenofibrate on HF have rarely been supported by the other studies.

Therefore, this study aimed to evaluate the association between fenofibrate use and HF outcomes in people with T2D within a real-world setting. We investigated the risk of HF-related outcomes between individuals receiving statin plus fenofibrate vs. statin only.

## Methods

### Data source

We used the Korean National Health Insurance Service (NHIS) Customized Database, which includes nearly all citizens living in South Korea. The database contains longitudinal information (2005–22) on individuals’ demographics; disease records according to the International Classification of Diseases, Tenth Revision (ICD-10); prescription records; medical and surgical procedures; hospitalizations; health examination data, including anthropometric measures and laboratory data; and death records. Details of the cohort protocol have been described previously.^21-26^

This study was approved by the Institutional Review Board of Korea University Anam Hospital (IRB number: 2020AN0099). The NHIS waived the requirement for informed consent because the database did not contain personally identifiable information. This study was conducted as part of the Effectiveness of Fenofibrate Therapy in Residual Cardiovascular Risk Reduction in the Real-World Setting (ECLIPSE-REAL) study.

### Study participant selection and propensity score matching

**Figure 1:**
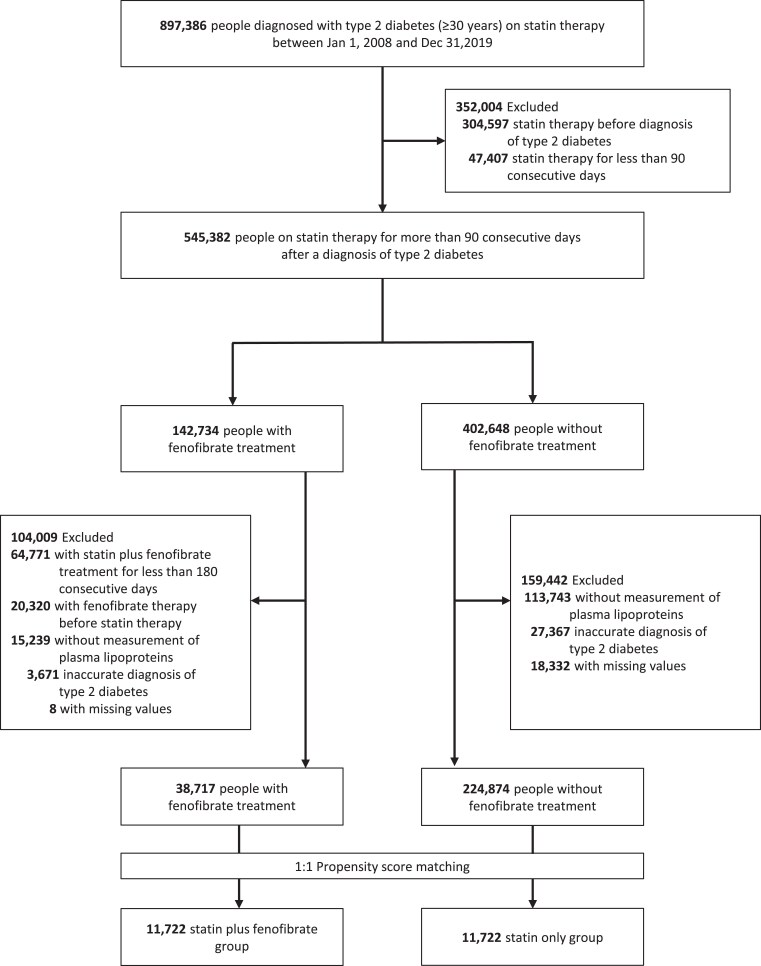
Participant selection process.

*Figure 1* illustrates the participant selection process. From 1 January 2008, to 31 December 2019, participants diagnosed with T2D (≥30 years of age) receiving statin therapy were selected (*n* = 897 386). Type 2 diabetes was defined based on ICD-10 code E11 plus anti-diabetic drugs. Individuals who received statin therapy before the diagnosis of T2D (*n* = 304 597) or those who received statin therapy for <90 consecutive days (*n* = 47 407) were excluded. We classified the patients into two groups based on the use of fenofibrate: (i) the statin plus fenofibrate group and (ii) statin only group. Those who received fenofibrate for <180 consecutive days (*n* = 64 771) or those who received fenofibrate before statin therapy (*n* = 20 320) were excluded from the statin plus fenofibrate group. In both groups, those who had no data on plasma lipoproteins or variables for propensity score (PS) matching and those who also received the E10 code (diagnosis of Type 1 diabetes) were excluded. Among the 38 717 participants in the statin plus fenofibrate treatment group and 224 874 participants in the statin only group, 1:1 PS matching was performed. The PS was calculated at the index date.

The index date was set as the start date of fenofibrate treatment in the statin plus fenofibrate group. To avoid the immortal time bias, the following process was performed to define the index date for the statin only group. We matched the distribution of the statin monotherapy period before enrolment between groups by identifying individuals who were treated with statins only at the index date of the statin plus fenofibrate group using the risk set sampling method with replacement. The index date for participants in the statin only group was set as the index date of the matched participants in the statin plus fenofibrate treatment group.

PS matching (1:1) was performed for the statin plus fenofibrate and statin only groups. We derived a PS model from multiple logistic regression that included age, sex, duration of diabetes, body mass index (BMI), waist circumference, fasting glucose, systolic blood pressure, estimated glomerular filtration rate (eGFR), duration of statin therapy, LDL-C, HDL-C, triglycerides, income level, smoking status, alcohol consumption, physical activity, pre-existing ischaemic heart disease, pre-existing ischaemic stroke, pre-existing HF, use of antithrombotic agents, anti-hypertensive agents, anti-diabetic agents, renin–angiotensin–aldosterone system (RAAS) inhibitors, sodium–glucose co-transporter-2 (SGLT2) inhibitors, glucagon-like peptide-1 receptor agonists, and statin intensity. We used greedy nearest neighbour matching on the logit of the PS with a calliper width of 0.2 of the standard deviation (SD) of the logit. An absolute standardized mean difference (SMD) of <0.05 was considered an acceptable balance of covariates between groups. Variables with an absolute SMD ≥0.05 were adjusted for the outcome analyses. Finally, 23 444 participants (11 722 in the statin plus fenofibrate group and 11 722 in the statin only group) were included in the analyses.

### Outcome measures

The primary outcomes were HHF and a composite of HHF or death from cardiovascular causes. Hospitalization for heart failure was defined as receiving an ICD-10 code of I50 (HF) with hospitalization through the emergency department or the use of intravenous loop diuretics. Death from cardiovascular causes was defined as death from cardiovascular disease (ICD codes I00–I99).

Secondary outcomes included acute kidney injury (AKI), end-stage kidney disease (ESKD), and death from any cause. Acute kidney injury was defined as newly diagnosed ICD-10 code N17 after the index date. End-stage kidney disease events were defined using ICD-10 code N18 or dialysis procedure codes and special exemption codes for V001 haemodialysis, V003 peritoneal dialysis, and V005 kidney transplantation. Additionally, we investigated changes in lipid parameters, including total cholesterol, LDL-C, HDL-C, and triglycerides.

Each participant was followed for up to 5 years from the index date until the earliest occurrence of the primary outcome, death, or end of the study period (31 December 2022).

### Statistical analysis

Baseline characteristics were compared between the groups by calculating the SMD. Variables with an absolute SMD ≥0.05 were adjusted for the outcome analyses. The incidence rates of the outcomes were computed as the number of events per 1000 person-years. A Cox proportional hazards regression model with a robust sandwich variance estimator was used to investigate the association between the treatment and study outcomes, considering the correlations within matched pairs. The risks of outcomes in the statin plus fenofibrate group were compared with those in the statin only group and presented as adjusted hazard ratios (HRs) and 95% confidence intervals (CIs). We also stratified the analyses by the presence or absence of pre-existing HF. Sensitivity analyses were conducted among individuals with ≥80% adherence. Adherence was defined as the sum of the prescription period divided by the total follow-up period.

Subgroup analyses were done according to baseline characteristics of participants including age (<65 vs. ≥65 years), sex (female vs. male), fasting glucose (<126 vs. ≥126 mg/dL), triglycerides (<200 vs. ≥200 mg/dL), HDL-C (<40 vs. ≥40 mg/dL), eGFR (<60 vs. ≥60 mL/min/1.73 m^2^), pre-existing HF (yes or no), pre-existing atherosclerotic cardiovascular disease (yes or no), hypertension (yes or no), and statin intensity (low, moderate, or high). Pre-existing atherosclerotic cardiovascular disease was defined as pre-existing ischaemic heart disease (ICD-10 codes I20–I25) or ischaemic stroke (ICD-10 codes I63–I64).

All statistical analyses were performed using the SAS Enterprise Guide software, version 7.1 (SAS Institute Inc., Cary, NC, USA) and a two-sided *P*-value <0.05 was considered statistically significant.

## Results

### Study population baseline characteristics

*Table 1* presents the baseline characteristics of the study participants before and after PS matching. Prior to PS matching, most variables differed between the groups. Participants in the statin plus fenofibrate group were younger, had a higher BMI, and had markedly higher triglyceride levels. After PS matching, most variables were well balanced between the groups. The mean age (SD) was 58.2 (10.3) years, mean BMI was 26.2 (3.5) kg/m^2^, and 65.6% were men. The prior histories of ischaemic heart disease (27.4%), ischaemic stroke (9.0%), and HF (7.4%) were well balanced. Moderate-intensity statins were used in 92.1% of patients. Variables that were not balanced after PS matching, such as LDL-C categories, triglyceride levels, and duration of statin use, were further adjusted in the outcome analyses. The median [interquartile range (IQR)] triglyceride levels were 158.0 (112.0, 219.0) and 165.0 (116.0, 233.0) mg/dL in the statin only and statin plus fenofibrate group.

**Table 1:** Baseline characteristics before and after propensity score matching

	Before matching			After matching		
	Statin only (*N* = 156 897)	Statin plus fenofibrate (*N* = 35 985)	SMD	Statin only (*N* = 11 722)	Statin plus fenofibrate (*N* = 11 722)	SMD
Age, years	59.9 (11.4)	56.0 (10.5)	−0.359	58.2 (10.4)	58.1 (10.1)	−0.001
Male, *n* (%)	90 020 (57.4)	25 167 (69.9)	0.263	7659 (65.3)	7721 (65.9)	0.011
Duration of diabetes, months	48.9 (38.1)	69.0 (41.5)	0.507	77.6 (34.8)	79.2 (35.6)	0.044
BMI, kg/m²	25.5 (3.5)	26.4 (3.5)	0.257	26.2 (3.6)	26.2 (3.4)	<0.001
Waist circumference, cm	86.2 (8.9)	88.8 (8.9)	0.292	88.0 (9.1)	88.1 (8.7)	0.014
Fasting glucose, mg/dL	133.1 (40.6)	144.2 (47.2)	0.252	140.0 (42.5)	140.5 (44.7)	0.012
Systolic BP, mmHg	126.6 (14.7)	127.1 (14.1)	0.033	126.7 (14.2)	126.9 (14.1)	0.009
eGFR, mL/min/1.73 m^2^	88.1 (25.1)	89.6 (26.4)	0.058	88.7 (22.8)	88.8 (27.6)	0.002
Duration of statin use, months	11.5 (15.8)	37.4 (30.9)	1.053	47.7 (22.0)	49.7 (24.0)	0.086
LDL-C, mg/dL	87.6 (39.4)	86.9 (46.7)	−0.015	86.7 (40.2)	86.7 (46.2)	<0.001
<100, *n* (%)	26 585 (16.9)	13 254 (36.8)	0.054	8135 (69.4)	8098 (69.1)	0.053
100–129, *n* (%)	6044 (3.9)	3548 (9.9)		2027 (17.3)	2053 (17.5)	
130–159, *n* (%)	3304 (2.1)	1756 (4.9)		1021 (8.7)	1055 (9.0)	
≥160, *n* (%)	1993 (1.3)	922 (2.6)		539 (4.6)	516 (4.4)	
HDL-C, mg/dL	51.3 (15.3)	47.6 (14.0)	−0.253	48.8 (12.3)	48.7 (13.8)	−0.011
<40, *n* (%)	6219 (4.0)	4940 (13.7)	0.305	2527 (21.6)	2599 (22.2)	<0.001
40–49, *n* (%)	12 701 (8.1)	7343 (20.4)		4343 (37.0)	4324 (36.9)	
≥50, *n* (%)	19 074 (12.2)	7284 (20.2)		4852 (41.4)	4799 (40.9)	
Triglycerides, mg/dL, median (IQR)	115.0 (83.0, 161.0)	192.0 (131.0, 283.0)	0.682	158.0 (112.0, 219.0)	165.0 (116.0, 233.0)	0.085
<100, *n* (%)	14 249 (9.1)	2419 (6.7)	0.916	1979 (16.9)	1933 (16.5)	0.085
100–149, *n* (%)	12 137 (7.7)	4113 (11.4)		3287 (28.0)	3096 (26.4)	
150–199, *n* (%)	6205 (4.0)	3769 (10.5)		2649 (22.6)	2554 (21.8)	
≥200, *n* (%)	5403 (3.4)	9266 (25.7)		3807 (32.5)	4139 (35.3)	
Individual income, *n* (%)			0.081			<0.001
Low	38 654 (24.6)	9414 (26.2)		2916 (24.9)	2915 (24.9)	
Middle	45 105 (28.7)	10 848 (30.1)		3519 (30.0)	3487 (29.7)	
High	70 270 (44.8)	15 174 (42.2)		5287 (45.1)	5320 (45.4)	
Smoking, *n* (%)						0.024
Never	19 690 (12.5)	7359 (20.5)		5463 (46.6)	5363 (45.8)	
Former	8264 (5.3)	4201 (11.7)		2800 (23.9)	2841 (24.2)	
Current	7489 (4.8)	5817 (16.2)		3459 (29.5)	3518 (30.0)	
Alcohol consumption, *n* (%)			0.286			0.028
Never	21 762 (13.9)	8151 (22.7)		6036 (51.5)	5907 (50.4)	
1–2 times/week	9318 (5.9)	5823 (16.2)		3711 (31.7)	3764 (32.1)	
≥3 times/week	4349 (2.8)	3398 (9.4)		1975 (16.8)	2051 (17.5)	
Physical activity, *n* (%)			0.076			0.025
Never	7608 (4.8)	3715 (10.3)		2500 (21.3)	2537 (21.6)	
1–2 times/week	7171 (4.6)	3982 (11.1)		2566 (21.9)	2565 (21.9)	
≥3 times/week	20 664 (13.2)	9681 (26.9)		6656 (56.8)	6620 (56.5)	
Comorbidities, *n* (%)						
Ischaemic heart disease	40 407 (25.8)	8589 (23.9)	−0.044	3199 (27.3)	3229 (27.5)	0.006
Ischaemic stroke	17 395 (11.1)	2816 (7.8)	−0.112	1031 (8.8)	1079 (9.2)	0.014
Heart failure	12 130 (7.7)	2385 (6.6)	−0.043	859 (7.3)	868 (7.4)	0.003
Antithrombotic agents, *n* (%)	45 536 (29.0)	9162 (25.5)	−0.080	3360 (28.7)	3379 (28.8)	0.004
Anti-hypertensive drugs, *n* (%)	77 458 (49.4)	18 666 (51.9)	0.050	6274 (53.5)	6291 (53.7)	0.003
Anti-diabetic drugs, *n* (%)	114 744 (73.1)	30 648 (85.2)	0.300	9926 (84.7)	9893 (84.4)	−0.008
RAAS inhibitors, *n* (%)	59 495 (37.9)	15 850 (44.0)	0.125	5222 (44.5)	5248 (44.8)	0.004
SGLT2 inhibitors, *n* (%)	6855 (4.4)	3853 (10.7)	0.242	995 (8.5)	1019 (8.7)	0.007
GLP-1RAs, *n* (%)	57 (0.0)	113 (0.3)	0.066	9 (0.1)	13 (0.1)	0.011
Statin intensity, *n* (%)			0.048			<0.001
Low	4888 (3.1)	1178 (3.3)		454 (3.9)	476 (4.1)	
Moderate	143 828 (91.7)	33 449 (93.0)		10 816 (92.3)	10 781 (92.0)	
High	8181 (5.2)	1358 (3.8)		452 (3.9)	465 (4.0)	

Data are presented as mean (standard deviation) or median (IQR) unless otherwise indicated.

BMI, body mass index; BP, blood pressure; eGFR, estimated glomerular filtration rate; GLP-1RA, glucagon-like peptide-1 receptor agonist; HDL-C, HDL cholesterol; IQR, interquartile range; LDL-C, LDL cholesterol; RAAS, renin–angiotensin–aldosterone system; SGLT2, sodium–glucose co-transporter-2; SMD, standardized mean difference; WC, waist circumference.

### Heart failure outcomes

During a median follow-up of 50.4 months (IQR 36.0, 60.0), HHF occurred in 199 of 11 722 statin only users (incidence rate: 4.13 per 1000 person-years) and 160 of 11 722 statin plus fenofibrate users (incidence rate: 3.44 per 1000 person-years), indicating a lower risk of HHF in the statin plus fenofibrate group compared with the statin only group [adjusted HR, 0.80 (95% CI, 0.65–0.98); *P* = 0.036] (*Table 2*). The statin plus fenofibrate group also exhibited a significantly lower risk of the composite outcome of HHF or cardiovascular death (adjusted HR, 0.79; 95% CI, 0.65–0.96; *P* = 0.016). The adjusted HR for cardiovascular death was 0.72 (95% CI, 0.48–1.07; *P* = 0.101), and the adjusted HR for all-cause mortality was 0.73 (95% CI, 0.62–0.86; *P* < 0.001).

**Table 2:** Risk of heart failure outcomes and secondary outcomes by treatment group

	Statin only (*n* = 11 722)		Statin plus fenofibrate (n = 11 722)			
	No. of events	Incidence rate^a^	No. of events	Incidence rate^a^	Adjusted^b^ HR (95% CI)	*P*-value
Primary analysis						
HHF	199	4.13	160	3.44	0.80 (0.65–0.98)	0.036
Composite HF outcome (HHF or CV death)	245	5.10	195	4.19	0.79 (0.65–0.96)	0.016
CV death	64	1.32	46	0.98	0.72 (0.48–1.07)	0.101
Sensitivity analysis (≥80% adherence)						
HHF	128	4.04	35	2.83	0.63 (0.43–0.92)	0.017
Composite HF outcome (HHF or CV death)	146	4.61	43	3.48	0.68 (0.48–0.97)	0.032
CV death	28	0.88	10	0.80	0.87 (0.40–1.88)	0.725
Secondary outcomes						
AKI	196	4.07	253	5.45	1.32 (1.09–1.59)	0.004
ESKD	26	0.54	19	0.41	0.76 (0.42–1.38)	0.369
Death from any cause	355	7.41	255	5.49	0.73 (0.62–0.86)	<0.001

AKI, acute kidney injury; CI, confidence interval; CV, cardiovascular; ESKD, end-stage kidney disease; HF, heart failure; HHF, hospitalization for HF; HR, hazard ratio.

^a^Incidence rate per 1000 person-years.

^b^Adjusted for variables with absolute SMD ≥0.05 between two groups (LDL cholesterol categories, triglycerides, and duration of statin use).

In sensitivity analyses restricted to individuals with ≥80% adherence, fenofibrate use was also associated with a significantly lower risk of HHF (adjusted HR, 0.63; 95% CI, 0.43–0.92; *P* = 0.017) and the composite outcome of HHF or cardiovascular death (adjusted HR, 0.68; 95% CI, 0.48–0.97; *P* = 0.032).

We also stratified the analyses by the presence or absence of pre-existing HF (see Supplementary material online, *Table S1* and *Figures S1* and *S2*). Among individuals without pre-existing HF, the adjusted HR for HHF was 0.78 (95% CI, 0.59–1.05; *P* = 0.102) in the primary analysis and 0.52 (95% CI, 0.29–0.95; *P* = 0.032) in the sensitivity analysis restricted to individuals with ≥80% adherence. Among those with pre-existing HF, the adjusted HR was 0.81 (95% CI, 0.60–1.10; *P* = 0.173) in the primary analysis and 0.67 (95% CI, 0.41–1.11; *P* = 0.122) in the sensitivity analysis. However, no significant interaction was observed between groups (*P* for interaction = 0.726). Fenofibrate use was associated with a lower risk of composite outcome of HHF or cardiovascular death, particularly among individuals with pre-existing HF; however, the interaction according to the presence or absence of pre-existing HF was not statistically significant (*P* for interaction = 0.657).

**Figure 2:**
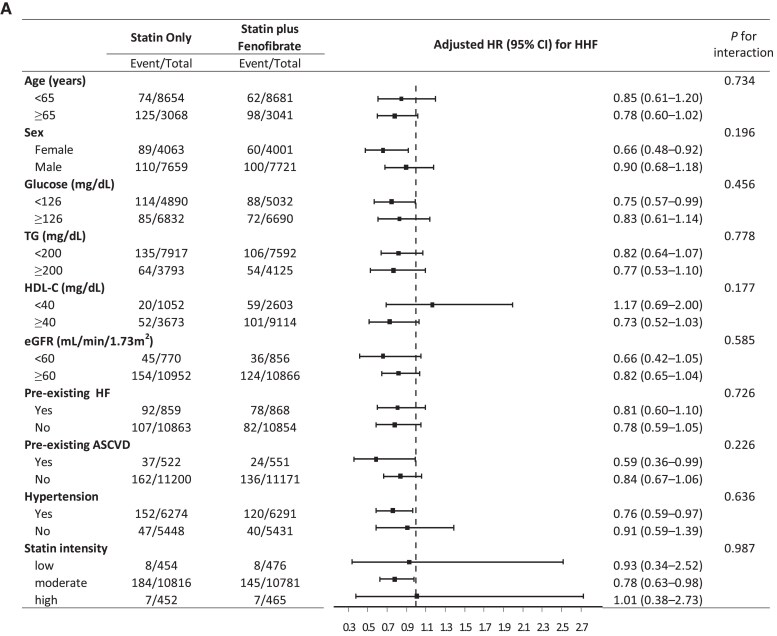

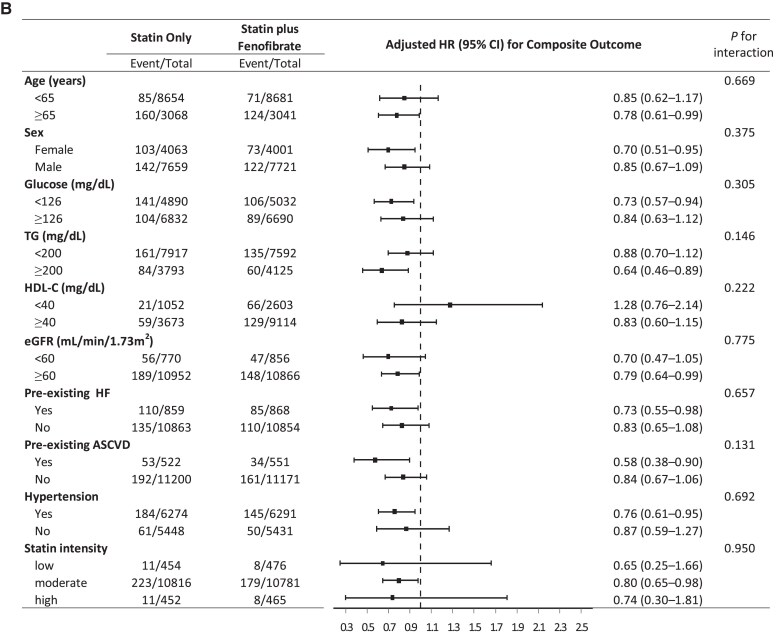
Subgroup analyses for (*A*) hospitalization for heart failure and (*B*) the composite outcome of hospitalization for heart failure or cardiovascular death. ASCVD, atherosclerotic cardiovascular disease; CI, confidence interval; eGFR, estimated glomerular filtration rate; HDL-C, HDL cholesterol; HF, heart failure; HR, hazard ratio; TG, triglycerides.

A subgroup analysis was performed to determine whether differences existed in the benefit of fenofibrate depending on patient characteristics (*Figure 2*). The reduced risk of HHF and the composite outcome of HHF and cardiovascular death in the statin plus fenofibrate group vs. the statin only group did not differ by patient characteristics (*P* for interaction >0.05 for all).

### Kidney outcomes

The incidence rates for AKI were 4.07 and 5.45 per 1000 person-years in the statin only and statin plus fenofibrate groups, respectively (adjusted HR, 1.32; 95% CI, 1.09–1.59; *P* = 0.004) (*Table 2*). In contrast, events corresponding to ESKD occurred in 26 of 11 722 patients in the statin only group (incidence rate: 0.54 per 1000 person-years) and in 19 of 11 722 patients in the statin plus fenofibrate group (incidence rate: 0.41 per 1000 person-years). The adjusted HR of developing ESKD was 0.76 (95% CI, 0.42–1.38; *P* = 0.369).

## Discussion

In this cohort study, the use of fenofibrate was associated with reduced risks of HHF and composite of HHF or cardiovascular death in patients with T2D treated with statins. Compared with non-users of fenofibrate (statin only group), fenofibrate users (statin plus fenofibrate group) exhibited a 20% lower risk of HHF and a 21% lower risk of the composite outcome of HHF or cardiovascular death. This beneficial effect of fenofibrate on HF outcomes was consistent with the sensitivity analysis restricted to individuals with ≥80% adherence. To the best of our knowledge, this is the first and largest real-world clinical study addressing benefits of fenofibrate for HF.

Randomized clinical trials of fenofibrate, including the Fenofibrate Intervention and Event Lowering in Diabetes (FIELD) and Action to Control Cardiovascular Risk in Diabetes (ACCORD) Lipid studies have yielded mixed results regarding the cardiovascular effects of fenofibrate.^27,28^ Of note, in the ACCORD Lipid study, the combination of simvastatin and fenofibrate was not superior to simvastatin alone for reducing the risk of major adverse cardiovascular events in patients with T2D. Subgroup analysis of the FIELD and ACCORD studies, along with a meta-analysis of clinical trials of fibrate drugs, indicated that fenofibrate might be beneficial only for specific subgroups of patients with T2D, particularly those with atherogenic dyslipidaemia.^29-31^

Recently, however, a post-hoc analysis of the ACCORD study suggested a different kind of beneficial cardiovascular effect of fenofibrate, specifically on HF-related outcomes; fenofibrate reduced the composite outcome of HHF or cardiovascular death compared with placebo (6.9% vs. 8.3%: HR, 0.82, 95% CI, 0.68–1.00, *P* = 0.048).^20^ This effect was more pronounced in patients with standard glucose-lowering therapy, in whom fenofibrate significantly reduced the composite outcome by 36% and HHF by 40%. Similarly, a combined analysis of the ACCORD Lipid and Veterans Affairs HDL Intervention Trial (VA-HIT) showed that fibrates were associated with an 18% reduction in the risk of HHF.^30^ Multiple animal and pre-clinical studies support the mechanistic linkage between fibrates and the prevention of hypertensive heart damage, cardiac hypertrophy, and HF. This effect is thought to be partly mediated by activation of PPAR-α, which has been linked to various cardioprotective effects.^32^ Fenofibrate has also been found to alleviate HF by modulating fatty acid metabolism, which is often disrupted in HF.^33^ Additionally, fenofibrate has been shown to ameliorate endothelial expression of cell adhesion molecules, which are involved in the pathogenesis of HF.^34^

Our study was conducted in a clinical setting similar to the ACCORD Lipid Trial. We targeted patients with T2D who received statin therapy at baseline. Our study demonstrated a clear beneficial effect of fenofibrate on HF-related outcomes, including both HHF and the composite outcome. The reduction in HF-related outcomes was greater in the sensitivity analysis among individuals with ≥80% adherence, which demonstrated that the use of fenofibrate was associated with a 37% lower risk of HHF and a 32% lower risk of composite outcome of HHF or cardiovascular death. Previous clinical studies of fibrates suggested that their effect on HF is independent of their lipid-modifying properties,^20^ while pre-clinical evidence indicated that an increase in fatty acid beta-oxidation via PPAR activation may contribute to preserving heart function.^35^ In our study, the combination of statin and fenofibrate significantly reduced triglyceride levels more than statin alone (see Supplementary material online, *Table S2*, −48.4 vs. −28.9 mg/dL; *P* < 0.001). In the subgroup analysis, the benefit of fenofibrate for the composite outcome of HHF and cardiovascular death was numerically greater in patients with triglyceride levels ≥200 mg/dL than in those with triglyceride levels <200 mg/dL, although the interaction between the groups was not significant (*P* for interaction = 0.146). This suggests that the lipid-modifying effects of fenofibrate may have influenced HF-related outcomes. In addition, in the ACCORD study, ∼60% of the participants were taking statins before study participation, and the remaining 40% were administered simvastatin at study entry. In contrast, all individuals in our study were on statin therapy before the index date, resulting in lower baseline LDL-C levels (86.7 vs. 100.6 mg/dL) than those in the ACCORD study. Fenofibrate is typically used as an add-on to statins in clinical practice; therefore, our study more closely replicates a realistic clinical scenario.

Fenofibrate treatment often results in increased serum creatinine levels and reduced eGFR.^36^ The increase in creatinine is often reversible upon discontinuation of the drug.^37,38^ A previous study suggested that fenofibrate does not change the actual glomerular filtration rate in patients with mild renal insufficiency, despite increasing blood creatinine.^39^ In contrast, fenofibrate showed beneficial renal effects including lower rates of incident albuminuria in multiple clinical trials.^40,41^ Therefore, actual renal effects of fenofibrate need to be evaluated in long-term observations. A post-hoc analysis of the ACCORD study showed that fenofibrate slowed long-term eGFR decline compared with placebo, although it caused a more transient increase in serum creatinine levels.^40^ In the current study, the risk of developing ESKD did not differ significantly between the statin plus fenofibrate group and the statin only groups (adjusted HR, 0.76; 95% CI, 0.42–1.38; *P* = 0.369).

### Limitations

This study had several limitations. First, its retrospective design means causal relationships could not be addressed. Second, although this study balanced the comparison groups, statins only vs. statins plus fenofibrate, by PS matching for a wide range of variables and adjusting for variables with an absolute SMD >0.05, the possibility of residual confounding due to unmeasured or unbalanced variables—such as medication adherence, healthcare access, and physician prescribing behaviours—cannot be ruled out. A selection bias may exist between the patients who were prescribed fenofibrate and those who were not, since adherence to lipid-lowering therapy or lifestyle factors could have contributed to the prescription of fenofibrate. Moreover, in individuals with pre-existing HF, the use of guideline-directed medical therapy may have skewed the findings. Third, we were unable to classify HF based on ejection fraction due to the lack of relevant data. Fourth, in retrospective pharmacoepidemiological studies, immortal time bias may exist. To reduce the probability of immortal time bias, we set the index date as the time taken to start fenofibrate in the control group. Fifth, this study primarily addressed HF outcomes; therefore, the analysis of kidney outcomes may have been biased by unmatched variables. A different analysis may be required for a detailed analysis of kidney outcomes. Additionally, data on albuminuria were not available, and future studies are warranted to investigate the effects of fenofibrate on albuminuria.

## Conclusions

In this real-world cohort study, the addition of fenofibrate to ongoing statin therapy was associated with reduced risks of HHF and the composite outcome of HHF and cardiovascular death in patients with T2D. Considering that HF is becoming an increasingly important complication and clinical outcome in patients with T2D, these results may support the use of fenofibrate as a therapeutic option for HF. Further investigations are required to validate the beneficial cardiovascular effects of fenofibrate.

## Supplementary Material

pvaf053_Supplementary_Data

## Data Availability

The data underlying this article will be shared on reasonable request to the corresponding author.

## References

[1] Jia G, Hill MA, Sowers JR. Diabetic cardiomyopathy: an update of mechanisms contributing to this clinical entity. Circ Res 2018;122:624–638. 29449364 10.1161/CIRCRESAHA.117.311586 PMC5819359

[2] Kim JH, Lee J, Han K, Kim J-T, Kwon H-S. Cardiovascular disease & diabetes statistics in Korea: nationwide data 2010 to 2019. Diabetes Metab J 2024;48:1084–1092. 10.4093/dmj.2024.0275 39610135 PMC11621650

[3] Moon JS, Kang S, Choi JH, Lee KA, Moon JH, Chon S, Kim DJ, Kim HJ, Seo JA, Kim MK, Lim JH, Song YJ, Yang YS, Kim JH, Lee Y-B, Noh J, Hur KY, Park JS, Rhee SY, Kim HJ, Kim HM, Ko JH, Kim NH, Kim CH, Ahn J, Oh TJ, Kim S-K, Kim J, Han E, Jin S-M, Bae J, Jeon E, Kim JM, Kang SM, Park JH, Yun J-S, Cha B-S, Moon MK, Lee B-W. 2023 clinical practice guidelines for diabetes management in Korea: full version recommendation of the Korean Diabetes Association. Diabetes Metab J 2024;48:546–708. 10.4093/dmj.2024.0249 39091005 PMC11307112

[4] Kim JY, Kim NH. Initial combination therapy in type 2 diabetes. Endocrinol Metab (Seoul) 2024;39:23–32. 10.3803/EnM.2023.1816 38031401 PMC10901659

[5] Echouffo-Tcheugui JB, Xu H, DeVore AD, Schulte PJ, Butler J, Yancy CW, Bhatt DL, Hernandez AF, Heidenreich PA, Fonarow GC. Temporal trends and factors associated with diabetes mellitus among patients hospitalized with heart failure: findings from Get With the Guidelines-Heart Failure registry. Am Heart J 2016;182:9–20. 10.1016/j.ahj.2016.07.025 27914505

[6] Palazzuoli A, Iacoviello M. Diabetes leading to heart failure and heart failure leading to diabetes: epidemiological and clinical evidence. Heart Fail Rev 2023;28:585–596. 35522391 10.1007/s10741-022-10238-6 PMC10140137

[7] Ceriello A, Catrinoiu D, Chandramouli C, Cosentino F, Dombrowsky AC, Itzhak B, Lalic NM, Prattichizzo F, Schnell O, Seferović PM, Valensi P, Standl E. Heart failure in type 2 diabetes: current perspectives on screening, diagnosis and management. Cardiovasc Diabetol 2021;20:218. 34740359 10.1186/s12933-021-01408-1 PMC8571004

[8] Gollmer J, Zirlik A, Bugger H. Mitochondrial mechanisms in diabetic cardiomyopathy. Diabetes Metab J 2020;44:33–53. 32097997 10.4093/dmj.2019.0185 PMC7043970

[9] Kjekshus J, Apetrei E, Barrios V, Böhm M, Cleland JGF, Cornel JH, Dunselman P, Fonseca C, Goudev A, Grande P, Gullestad L, Hjalmarson Å, Hradec J, Jánosi A, Kamenský G, Komajda M, Korewicki J, Kuusi T, Mach F, Mareev V, McMurray JJV, Ranjith N, Schaufelberger M, Vanhaecke J, van Veldhuisen DJ, Waagstein F, Wedel H, Wikstrand J. Rosuvastatin in older patients with systolic heart failure. N Engl J Med 2007;357:2248–2261. 10.1056/NEJMoa0706201 17984166

[10] Anderson JL, May HT, Le VT, Muhlestein JB, Horne BD, Bair TL, Knight S, Knowlton KU. Impact of statin therapy in heart failure patients: results of a large real-world experience. JACC Adv 2023;2:100385. 10.1016/j.jacadv.2023.100385 38938227 PMC11198218

[11] Hirano T . Pathophysiology of diabetic dyslipidemia. J Atheroscler Thromb 2018;25:771–782. 29998913 10.5551/jat.RV17023 PMC6143775

[12] Won H, Bae JH, Lim H, Kang M, Kim M, Lee S-H. 2024 KSoLA consensus on secondary dyslipidemia. J Lipid Atheroscler 2024;13:215–231. 10.12997/jla.2024.13.3.215 39355405 PMC11439749

[13] Park K-Y, Hong S, Kim K-S, Han K, Park C-Y. Trends in prevalence of hypertriglyceridemia and related factors in Korean adults: a serial cross-sectional study. J Lipid Atheroscler 2023;12:201–212. 10.12997/jla.2023.12.2.201 37265850 PMC10232222

[14] Kim JY, Kim NH. New therapeutic approaches to the treatment of dyslipidemia 1: ApoC-III and ANGPTL3. J Lipid Atheroscler 2023;12:23–36. 10.12997/jla.2023.12.1.23 36761060 PMC9884553

[15] Kim NH, Lee J, Chon S, Yu JM, Jeong I-K, Lim S, Kim WJ, Song K, Cho HC, Yu HM, Kim K-A, Kim SS, Lee SH, Kim CH, Kwak SH, Lee Y, Chung CH, Lee S, Jin HY, Lee JH, Koh G, Kim S-Y, Kim J, Lee JH, Kim TN, Jeon HJ, Lee JH, Jeon J-H, Yoo HJ, Kim HK, Park H-K, Nam-Goong IS, Hong S, Ahn CW, Yu JH, Park JH, Park K-G, Park CH, Joung KH, Ryu O-H, Park KY, Hong E-G, Cha B-S, Won KC, Chung Y-S, Kim SG. Study design and protocol for a randomized controlled trial to assess long-term efficacy and safety of a triple combination of ezetimibe, fenofibrate, and moderate-intensity statin in patients with type 2 diabetes and modifiable cardiovascular risk factors (ENSEMBLE). Endocrinol Metab (Seoul) 2024;39:722–731. 10.3803/EnM.2024.1995 39174014 PMC11525705

[16] Varbo A, Nordestgaard BG. Nonfasting triglycerides, low-density lipoprotein cholesterol, and heart failure risk: two cohort studies of 113 554 individuals. Arterioscler Thromb Vasc Biol 2018;38:464–472. 10.1161/atvbaha.117.310269 29097364

[17] Ebong IA, Goff DC, Rodriguez CJ, Chen H, Sibley CT, Bertoni AG. Association of lipids with incident heart failure among adults with and without diabetes mellitus: Multiethnic Study of Atherosclerosis. Circ Heart Fail 2013;6:371–378. 23529112 10.1161/CIRCHEARTFAILURE.112.000093 PMC3991930

[18] Lee H-C, Lin Y-H. The pathogenic role of very low density lipoprotein on atrial remodeling in the metabolic syndrome. Int J Mol Sci 2020;21:891. 32019138 10.3390/ijms21030891 PMC7037013

[19] Deerochanawong C, Kim SG, Chang Y-C. Role of fenofibrate use in dyslipidemia and related comorbidities in the Asian population: a narrative review. Diabetes Metab J 2024;48:184–195. 10.4093/dmj.2023.0168 38273789 PMC10995494

[20] Ferreira JP, Vasques-Nóvoa F, Ferrão D, Saraiva F, Falcão-Pires I, Neves JS, Sharma A, Rossignol P, Zannad F, Leite-Moreira A. Fenofibrate and heart failure outcomes in patients with type 2 diabetes: analysis from ACCORD. Diabetes Care 2022;45:1584–1591. 35320363 10.2337/dc21-1977 PMC9274224

[21] Cho SW, Kim JH, Choi HS, Ahn HY, Kim MK, Rhee EJ. Big data research in the field of endocrine diseases using the Korean National Health Information Database. Endocrinol Metab (Seoul) 2023;38:10–24. 10.3803/EnM.2023.102 36758542 PMC10008661

[22] Kim NH, Han KH, Choi J, Lee J, Kim SG. Use of fenofibrate on cardiovascular outcomes in statin users with metabolic syndrome: propensity matched cohort study. BMJ 2019;366:l5125. 10.1136/bmj.l5125 31562117 PMC6763755

[23] Kim NH, Kim JY, Choi J, Kim SG. Associations of omega-3 fatty acids vs. fenofibrate with adverse cardiovascular outcomes in people with metabolic syndrome: propensity matched cohort study. Eur Heart J Cardiovasc Pharmacother 2024;10:118–127. 10.1093/ehjcvp/pvad090 38017618

[24] Kim JY, Lee J, Moon JH, Park SE, Ko S-H, Choi SH, Kim NH. Prevalence, incidence, and metabolic characteristics of young adults with type 2 diabetes Mellitus in South Korea (2010–2020). Diabetes Metab J 2025;49:172–182. 10.4093/dmj.2024.0826 40073905 PMC11960202

[25] Kim K-S, Hong S, Han K, Park C-Y. Clinical characteristics of patients with statin discontinuation in Korea: a nationwide population-based study. J Lipid Atheroscler 2024;13:41–52. 10.12997/jla.2024.13.1.41 38299165 PMC10825567

[26] Jeong S-M, Jung J-H, Yang Y-S, Kim W, Cho IY, Lee Y-B, Park K-Y, Nam GE, Han K. 2023 obesity fact sheet: prevalence of obesity and abdominal obesity in adults, adolescents, and children in Korea from 2012 to 2021. J Obes Metab Syndr 2024;33:27–35. 10.7570/jomes24012 38531533 PMC11000515

[27] Keech A, Simes RJ, Barter P, Best J, Scott R, Taskinen MR, Forder P, Pillai A, Davis T, Glasziou P, Drury P, Kesäniemi YA, Sullivan D, Hunt D, Colman P, d’Emden M, Whiting M, Ehnholm C, Laakso M; FIELD Study Investigators. Effects of long-term fenofibrate therapy on cardiovascular events in 9795 people with type 2 diabetes mellitus (the FIELD study): randomised controlled trial. Lancet 2005;366:1849–1861. 10.1016/s0140-6736(05)67667-2 16310551

[28] ACCORD Study Group; Ginsberg HN, Elam MB, Lovato LC, Crouse JR 3rd, Leiter LA, Linz P, Friedewald WT, Buse JB, Gerstein HC, Probstfield J, Grimm RH, Ismail-Beigi F, Bigger JT, Goff DC Jr, Cushman WC, Simons-Morton DG, Byington RP. Effects of combination lipid therapy in type 2 diabetes mellitus. N Engl J Med 2010;362:1563–1574. 20228404 10.1056/NEJMoa1001282 PMC2879499

[29] Sacks FM, Carey VJ, Fruchart JC. Combination lipid therapy in type 2 diabetes. N Engl J Med 2010;363:692–694; author reply 694–695. 10.1056/NEJMc1006407 20842772

[30] Jun M, Foote C, Lv J, Neal B, Patel A, Nicholls SJ, Grobbee DE, Cass A, Chalmers J, Perkovic V. Effects of fibrates on cardiovascular outcomes: a systematic review and meta-analysis. Lancet 2010;375:1875–1884. 10.1016/s0140-6736(10)60656-3 20462635

[31] Lee M, Saver JL, Towfighi A, Chow J, Ovbiagele B. Efficacy of fibrates for cardiovascular risk reduction in persons with atherogenic dyslipidemia: a meta-analysis. Atherosclerosis 2011;217:492–498. 10.1016/j.atherosclerosis.2011.04.020 21592479

[32] Balakumar P, Rohilla A, Mahadevan N. Pleiotropic actions of fenofibrate on the heart. Pharmacol Res 2011;63:8–12. 10.1016/j.phrs.2010.11.002 21093591

[33] Li P, Luo S, Pan C, Cheng X. Modulation of fatty acid metabolism is involved in the alleviation of isoproterenol-induced rat heart failure by fenofibrate. Mol Med Rep 2015;12:7899–7906. 26497978 10.3892/mmr.2015.4466 PMC4758294

[34] Yin WH, Chen JW, Chen YH, Lin SJ. Fenofibrate modulates HO-1 and ameliorates endothelial expression of cell adhesion molecules in systolic heart failure. Acta Cardiol Sin 2013;29:251–260. 27122714 PMC4804837

[35] Sarma S, Ardehali H, Gheorghiade M. Enhancing the metabolic substrate: PPAR-alpha agonists in heart failure. Heart Fail Rev 2012;17:35–43. 10.1007/s10741-010-9208-0 21104312

[36] Kostapanos MS, Florentin M, Elisaf MS. Fenofibrate and the kidney: an overview. Eur J Clin Invest 2013;43:522–531. 10.1111/eci.12068 23480615

[37] Davis TME, Ting R, Best JD, Donoghoe MW, Drury PL, Sullivan DR, Jenkins AJ, O’Connell RL, Whiting MJ, Glasziou PP, Simes RJ, Kesäniemi YA, Gebski VJ, Scott RS, Keech AC. Effects of fenofibrate on renal function in patients with type 2 diabetes mellitus: the Fenofibrate Intervention and Event Lowering in Diabetes (FIELD) study. Diabetologia 2011;54:280–290. 10.1007/s00125-010-1951-1 21052978

[38] Mychaleckyj JC, Craven T, Nayak U, Buse J, Crouse JR, Elam M, Kirchner K, Lorber D, Marcovina S, Sivitz W, Sperl-Hillen J, Bonds DE, Ginsberg HN. Reversibility of fenofibrate therapy-induced renal function impairment in ACCORD type 2 diabetic participants. Diabetes Care 2012;35:1008–1014. 22432114 10.2337/dc11-1811 PMC3329840

[39] Hottelart C, El Esper N, Rose F, Achard J-M, Fournier A. Fenofibrate increases creatininemia by increasing metabolic production of creatinine. Nephron 2002;92:536–541. 10.1159/000064083 12372935

[40] Frazier R, Mehta R, Cai X, Lee J, Napoli S, Craven T, Tuazon J, Safdi A, Scialla J, Susztak K, Isakova T. Associations of fenofibrate therapy with incidence and progression of CKD in patients with type 2 diabetes. Kidney Int Rep 2019;4:94–102. 30596172 10.1016/j.ekir.2018.09.006 PMC6308372

[41] Ansquer J-C, Foucher C, Rattier S, Taskinen M-R, Steiner G. Fenofibrate reduces progression to microalbuminuria over 3 years in a placebo-controlled study in type 2 diabetes: results from the Diabetes Atherosclerosis Intervention Study (DAIS). Am J Kidney Dis 2005;45:485–493. 10.1053/j.ajkd.2004.11.004 15754270

