# Supplementary material for: Fenofibrate therapy and risk of heart failure outcomes in patients with Type 2 diabetes: a propensity-matched cohort study
Source: Eur Heart J Cardiovasc Pharmacother. 2025 Jul 21;11(7):620–9. doi: 10.1093/ehjcvp/pvaf053 (PMC12582659; doi:10.1093/ehjcvp/pvaf053)
Supplement: pvaf053_Supplementary_Data [file pvaf053_supplementary_data.docx]

**Supplementary materials**

**Title: Fenofibrate therapy and risk of heart failure outcomes in patients with type 2 diabetes: a propensity-matched cohort study**

Ji Yoon Kim, Nam Hoon Kim, Jiyoon Lee, Dong-Hoon Kim, and Sin Gon Kim

**Supplementary Figure 1.** Adjusted risk of hospitalisation for HF in the total cohort (A), individuals without prior HF (B), and individuals with prior HF (C).

**Supplementary Figure 2.** Adjusted risk of composite heart failure (HF) outcome—hospitalisation for HF or cardiovascular death—in the total cohort (A), individuals without prior HF (B), and individuals with prior HF (C).

**Supplementary Table 1.** Risk of heart failure (HF) outcomes stratified by pre-existing HF status

**Supplementary Table 2.** Changes in lipid and lipoproteins

**Supplementary Figure 1.** Adjusted risk of hospitalisation for heart failure (HF) in the total cohort (A), individuals without prior HF (B), and individuals with prior HF (C).

(A) In total cohort


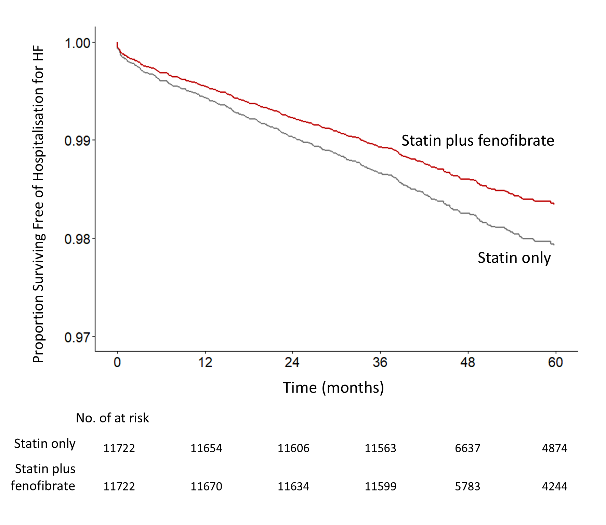


(B) Among individuals without prior HF


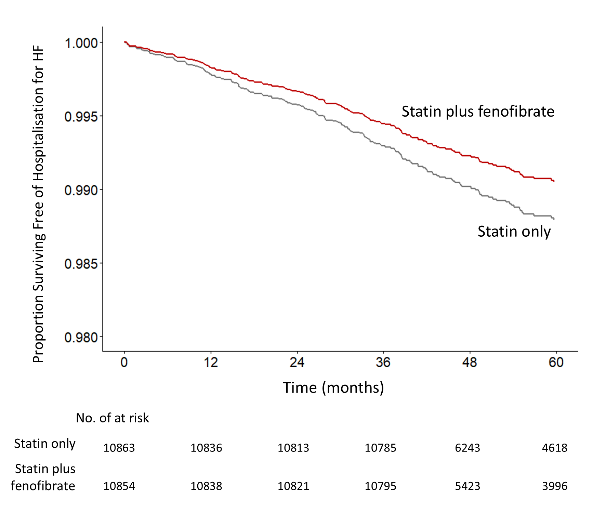


(C) Among individuals with prior HF


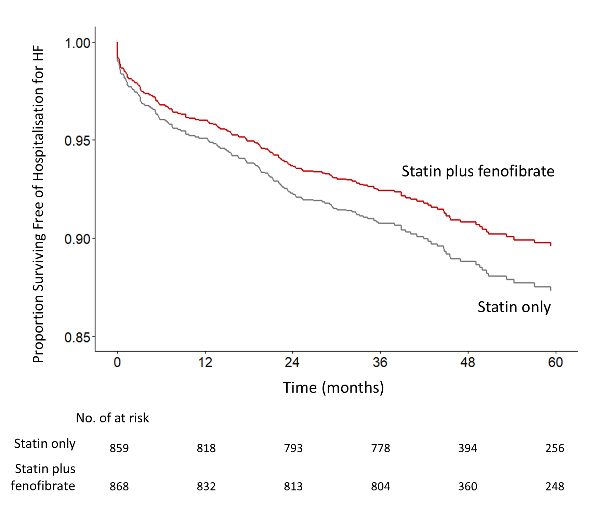


**Supplementary Figure 2.** Adjusted risk of composite heart failure (HF) outcome—hospitalisation for HF or cardiovascular death—in the total cohort (A), individuals without prior HF (B), and individuals with prior HF (C).

(A) In total cohort


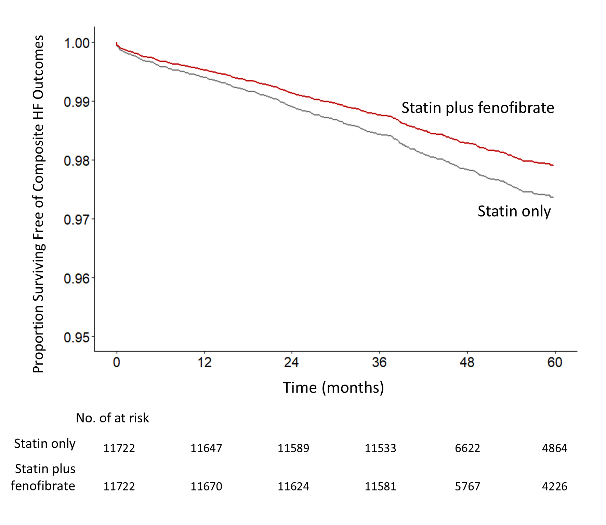


(B) Among individuals without prior HF


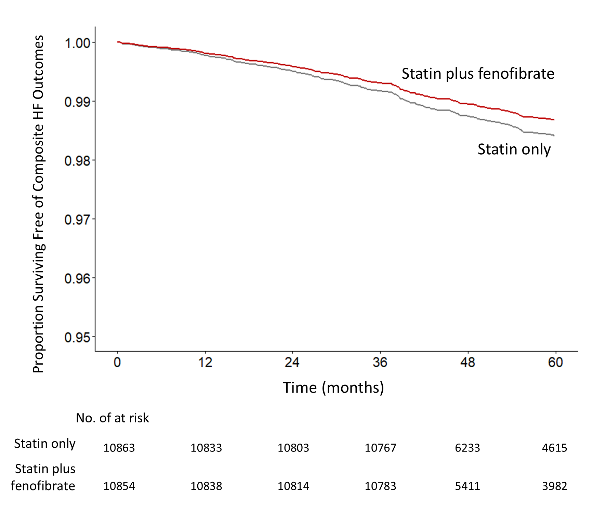


(C) Among individuals with prior HF


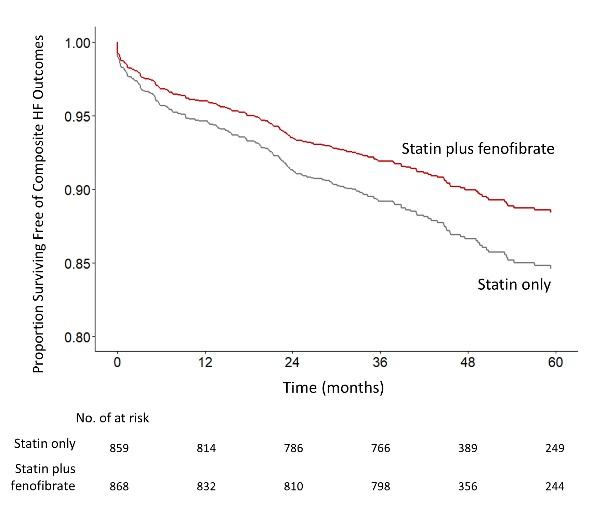


**Supplementary Table 1.** Risk of heart failure (HF) outcomes stratified by pre-existing HF status

|  | Statin only | | Statin plus fenofibrate | |  |  |
| --- | --- | --- | --- | --- | --- | --- |
|  | No. of events | Incidence rate* | No. of events | Incidence rate* | Adjusted** HR (95% CI) | *P*-value |
| In individuals without pre-existing HF (n=10863 for statin only group, n=10854 for statin plus fenofibrate group) | | | | | | |
| Primary analysis | | | | | | |
| HHF | 107 | 2.38 | 82 | 1.89 | 0.78 (0.59–1.05) | 0.102 |
| Composite HF outcome (HHF or CV death) | 135 | 3.01 | 11. | 2.54 | 0.83 (0.65–1.08) | 0.161 |
| CV death | 34 | 0.75 | 32 | 0.74 | 0.97 (0.58–1.61) | 0.903 |
| Sensitivity analysis (≥80% adherence) | | | | | | |
| HHF | 64 | 2.17 | 14 | 1.23 | 0.52 (0.29–0.95) | 0.032 |
| Composite HF outcome (HHF or CV death) | 73 | 2.48 | 20 | 1.75 | 0.66 (0.40–1.11) | 0.119 |
| CV death | 11 | 0.37 | 6 | 0.52 | 1.41 (0.46–4.32) | 0.541 |
| In individuals with pre-existing HF (n=859 for statin only group, n=868 for statin plus fenofibrate group) | | | | | | |
| Primary analysis |  |  |  |  |  |  |
| HHF | 92 | 28.74 | 78 | 24.50 | 0.81 (0.60–1.10) | 0.173 |
| Composite HF outcome (HHF or CV death) | 110 | 34.66 | 85 | 26.81 | 0.73 (0.55–0.98) | 0.035 |
| CV death | 30 | 8.84 | 14 | 4.16 | 0.44 (0.23–0.85) | 0.014 |
| Sensitivity analysis (≥80% adherence) | | | | | | |
| HHF | 64 | 28.56 | 21 | 21.98 | 0.67 (0.41–1.11) | 0.122 |
| Composite HF outcome (HHF or CV death) | 73 | 32.73 | 23 | 24.23 | 0.65 (0.40–1.05) | 0.076 |
| CV death | 17 | 7.17 | 4 | 3.98 | 0.50 (0.16–1.51) | 0.218 |

CI, confidence interval; CV, cardiovascular; HF, heart failure; HHF, hospitalisation for HF; HR, hazard ratio.

*Incidence rate per 1,000 person-years.

**Adjusted for variables with absolute SMD ≥0.05 between two groups (low-density lipoprotein cholesterol categories, triglycerides, and duration of statin use).

**Supplementary Table 2.** Changes in lipid and lipoproteins

|  | Statin only  (n=11722) | | | Statin plus fenofibrate  (n=11722) | | | *P*-value |
| --- | --- | --- | --- | --- | --- | --- | --- |
|  | At baseline | During treatment | Changes | At baseline | During treatment | Changes |  |
| TC, mg/dL  (mean (SD)) | 152.8 (31.3) | 147.0 (27.5) | −5.8 (30.6) | 154.9 (32.1) | 156.1 (64.5) | 1.3  (66.7) | <.001 |
| LDL-C, mg/dL  (mean (SD)) | 71.5 (26.6) | 69.5 (23.5) | −2.0 (27.0) | 71.1 (34.4) | 77.8 (62.5) | 6.7  (67.3) | <.001 |
| HDL-C, mg/dL  (mean (SD)) | 48.2 (11.6) | 49.5 (11.8) | 1.3 (10.0) | 48.1 (14.9) | 50.9 (14.6) | 2.8  (16.4) | 0.001 |
| TG, mg/dL  (mean (SD)) | 172.9 (99.4) | 144.0 (81.3) | −28.9 (95.6) | 190.3 (103.0) | 141.9 (84.2) | −48.4 (111.4) | <.001 |
| TG, mg/dL  (median (IQR)) | 153.0 (108.0, 213.0) | 127.0 (90.0, 176.0) | −21.0 (−65.0, 17.0) | 168.0 (117.0, 239.0) | 121.0 (85.0, 175.0) | −43.0 (−102.0, 10.0) |  |
| Non-HDL-C, mg/dL  (mean (SD)) | 104.7 (29.9) | 97.6 (25.9) | −7.1 (29.7) | 106.9 (30.7) | 105.1 (64.1) | −1.8 (66.2) | 0.005 |

Only patients whose lipid parameters were measured at baseline and after treatment were included in the analyses.

HDL-C, high-density lipoprotein cholesterol; LDL-C, low-density lipoprotein cholesterol; TC, total cholesterol; TG, triglycerides.
